# ﻿A new species in *Begonia* sect. *Diploclinium* (Begoniaceae) from Guangxi, South China

**DOI:** 10.3897/phytokeys.249.133724

**Published:** 2024-12-05

**Authors:** Ping-Ting Hu, Yu-Ni Huang, Meng-Ni Zhang, Bi-Xuan Chen, Shu-Shu Huang, Ren-Kun Li, Wen-Guang Wang, Xin-Xin Feng

**Affiliations:** 1 School of Urban Construction and Intelligent Manufacturing, Dongguan City University, Dongguan 523419, China Dongguan City University Dongguan China; 2 Dongguan Botanical Garden, Dongguan 523086, China Dongguan Botanical Garden Dongguan China; 3 Department of Horticulture and Landscape Architecture, Zhongkai University of Agriculture and Engineering, Guangzhou 510225, China Zhongkai University of Agriculture and Engineering Guangzhou China; 4 Enshi Gesneriad and Begonia Nursery Co., Ltd., Enshi 445000, China Enshi Gesneriad and Begonia Nursery Co., Ltd. Enshi China; 5 Xishuangbanna Tropical Botanical Garden, Chinese Academy of Sciences, Mengla, Yunnan, 666303, China Xishuangbanna Tropical Botanical Garden, Chinese Academy of Sciences Mengla China

**Keywords:** Limestone karst, morphology, new taxon, south China, taxonomy

## Abstract

Although Guangxi represents one of the distribution centres of begonias in China, the sect. Diploclinium (Wright) A. DC is not well documented herein. In this article, we illustrate a new species belonging to this section, *Begoniafangchengensis* Y.N.Huang, X.X. Feng & R.K.Li, which has been discovered in southern Guangxi. *Begoniafangchengensis* mostly resembles *B.rotundilimba* in elliptic leaf blade, palmate veins, dichasial cyme, three styles, axile placentation, and unequally 3-winged capsule. On the other hand, *B.fangchengensis* is characterized by creeping rhizome, pilose stipule, red and densely pilose petiole, pubescent leaf blade and pilose inflorescence bract, sparsely pilose ovary and the zygomorphic androecium, differing from the erect stem at anthesis, the green and densely villous petiole, the glabrous stipule, leaf blade, inflorescence bract, ovary and actinomorphic androecium in *B.rotundilimba*. Considering its small population size and narrow distribution, its conservation status is categorized as ‘Endangered (D)’ according to the IUCN Red List Categories and Criteria.

## ﻿Introduction

As one of the fastest-evolving genera in vascular plants around the world, *Begonia* L. species are now 2,151 in number (Hughes et al. 2015) and these are mainly distributed in the humid tropical and subtropical regions of Asia, America, and Africa. Over 270 species of *Begonia* have been reported in China and the taxa are expected to exceed 300 species in the future (Tian et al. 2018). The begonias are primarily distributed in southern and central China, with Yunnan and Guangxi representing the centers of natural distribution.

Approximately 90 species of *Begonia* have been documented in Guangxi and new species have been reported frequently in recent years (Liu et al. 2020; Feng et al. 2021; 2022; 2023; Tian et al. 2021; Zhou et al. 2024). The begonias in Guangxi are dominated by B.sect.Coelocentrum taxa from karst landforms. Only nine species belonging to the sect. Diploclinium are distributed in Guangxi, including: *Begoniabambusetorum* H. Q. Nguyen, Y. M. Shui & W. H. Chen, *Begoniafimbristipula* Hance, *Begoniagigabracteata* H. Z. Li & H. Ma, *Begoniaglechomifolia* C. M. Hu ex C. Y. Wu & T. C. Ku., *Begoniagrandis* Dryander, Begoniagrandissubsp.sinensis (A. Candolle) Irmscher, *Begoniahymenocarpa* C. Y. Wu, *Begoniaobsolescens* Irmscher and *Begoniasinovietnamica* C. Y. Wu.

In October 2021, an unknown begonia was discovered growing on the slope by a stream during fieldwork in the Shiwanshan Mountain, southern Guangxi. This taxon is distinct from the commonly karst distributed sect. Coelocentrum begonias in consideration of its ovary locule and placentae type in the field. Some individuals have been well cultivated in the nursery of Dongguan Botanical Garden and flower characters were observed in January 2022. Based on detailed morphological comparisons with similar species, this *Begonia* species is identified as a new species of the section Diploclinium.

## ﻿Materials and methods

Morphological characters were observed and measured from fresh samples in the field. Morphological comparisons with similar taxa were undertaken by consulting the literature, examining herbarium (**IBK** and **IBSC**) specimens and observing living collections cultivated in the nursery of Dongguan Botanical Garden. The specimens were deposited at the Herbarium of South China Botanical Garden (**IBSC**), CAS.

## ﻿Taxonomy

### ﻿ Begonia fangchengensis

Taxon classificationPlantaeCucurbitalesBegoniaceae

Y.N.Huang, X.X.Feng & R.K.Li sp. nov.

F36A060F-0BD8-57F8-B278-533F251D1453

urn:lsid:ipni.org:names:77346002-1

Figs 1 , 2

#### Type.

**China** • Guangxi Zhuang Autonomous Region (广西壮族自治区), Fangchenggang City (防城港市), Fangcheng District (防城区), Shiwanshan Yao Autonomous Town (十万山瑶族乡), Bajiao Mountain, 21°56'54"N, 108°14'20"E, (Fig. 3), 198 m alt., on the rock or slope beside the stream, 18 October 2021, *Yu Ni Huang, Xin Xin Feng* & *Ren Kun Li* (holotype: 0925761, IBSC!).

**Figure 1.**
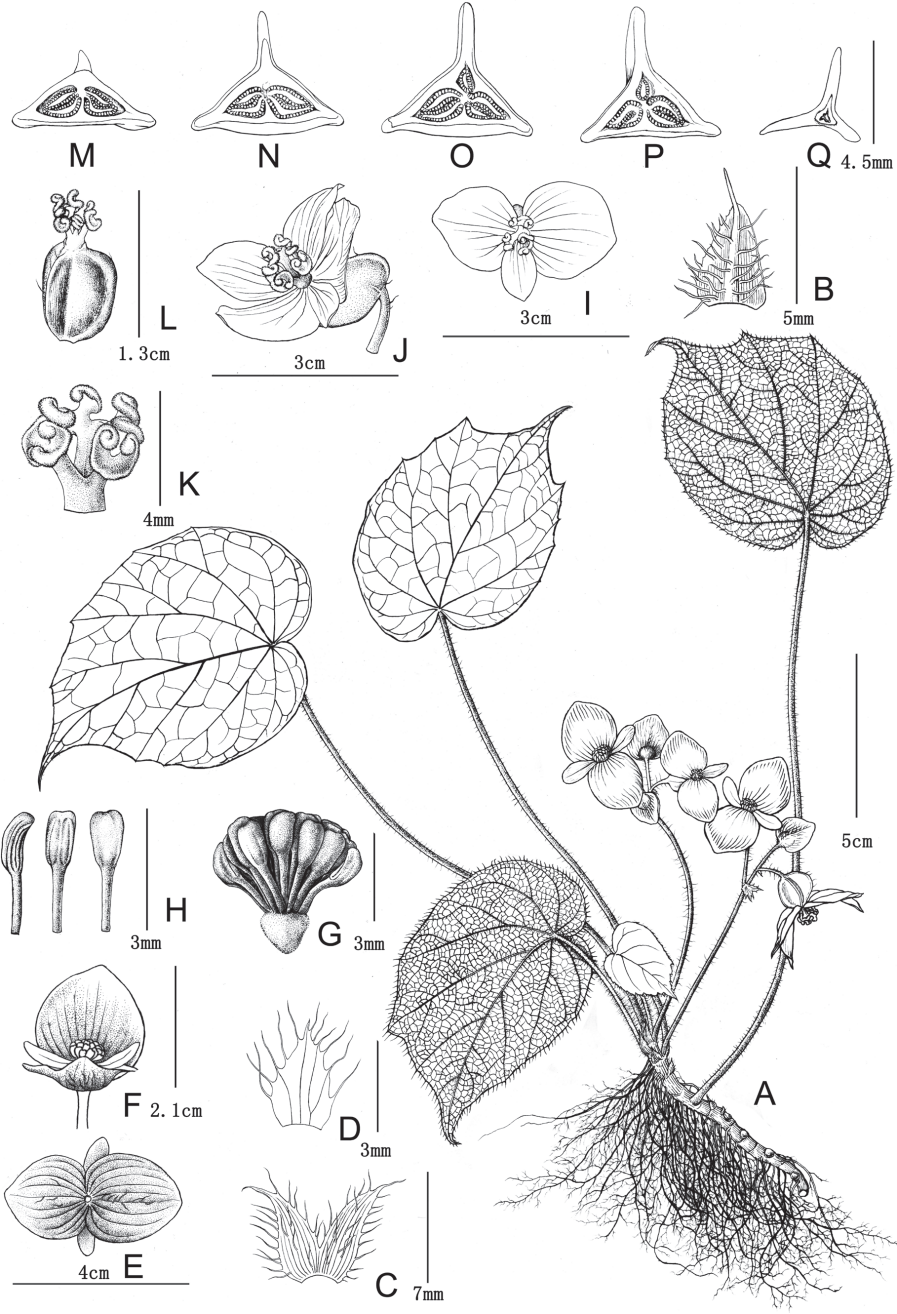
*Begoniafangchengensis* drawn by Yunxiao Liu **A** habitat **B** stipule **C, D** bracts of inflorescences **E, F** front and back view of staminate flower **G** androecium **H** stamens **I, J** pistillate flower with 3 or 5 tepals **K** styles **L** styles and ovary **M–Q** ovary section from upper to lower positions.

**Figure 2.**
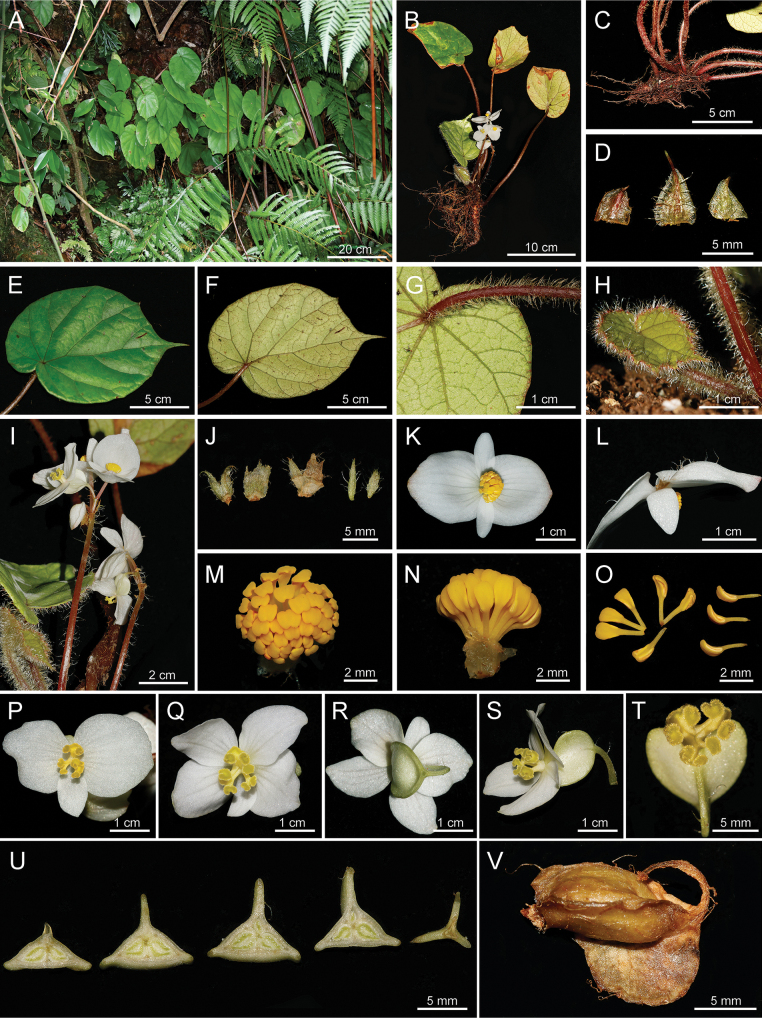
Habitat and morphology of *Begoniafangchengensis***A** habitat **B** flowering plant **C** creeping rhizome **D** stipules **E** leaf blade (adaxial) **F, G** leaf blade (abaxial) **H** juvenile leaf **I** inflorescence **J** bracts **K, L** front and side views of staminate flower **M, N** front and side views of androecium **O** stamens **P** pistillate flower with 3 tepals **Q, R, S** pistillate flower with 5 tepals **T** stigmas and ovary **U** ovary sections from upper to lower positions **V** capsule (Photos by Z.X. Liu).

#### Diagnosis.

*Begoniafangchengensis* mostly resembles *B.rotundilimba* in its elliptic leaf blade, palmate veins, dichasial cyme, three styles, axile placentation, and unequally 3-winged capsule. However, there are creeping rhizome, pilose stipule, red and densely pilose petiole, pubescent leaf blade and pilose inflorescence bract, sparsely pilose ovary and the zygomorphic androecium in *B.fangchengensis*. These characters differ from the erect stem at anthesis, the green and densely villous petiole, the glabrous stipule, leaf blade, inflorescence bract, ovary and actinomorphic androecium in *B.rotundilimba*.

#### Description.

Perennial evergreen herb, monoecious, 15–30 cm height.

***Rhizome*** creeping, ca. 6–8 cm long and 8–10 mm in diameter, internode short. ***Stipules*** membranous, reddish-green, translucent, triangular, 4.5–8 × 4–5 mm, pilose. ***Leaves*** all basal, petiole 10–15 cm long, red and densely white pilose; leave blade asymmetric, widely ovate to elliptic, 12–17 × 8–10 cm; fleshy; adaxially bright green and smooth; abaxially pale-green, densely covered with white pilose along the veins; base oblique-cordate; margin irregularly denticulate; apex caudate acuminate; venation palmate, primary veins 8, adaxially slightly concave, abaxially convex. ***Inflorescences*** arising directly from rhizome, dichasial cymes, peduncle 6.5–8 cm long, pilose; flowers unisexual, 2–5 flowers per inflorescence; bracts membranous, pale green, triangular to subcircular, 2–3 lobed apically, margin serrate with cilia, 7–8 × 4–6 mm. ***Staminate flower*** tepals 4, white; outer 2 larger, widely ovate, 17–21 × 15–20 mm, abaxially sparsely pubescent; inner 2, lanceolate, margin entire, 10–12 × 4–6 mm, glabrous; androecium zygomorphic, sub-globose, ca. 6 mm in diameter; stamens numerous, 3–3.5 mm long, filaments fused at base; anthers yellow, ca. 1.5 mm long, cuneiform, apex slightly bent and retuse. ***Pistillate flower*** tepals 3–5, white, glabrous; outer 2, widely ovate to subcircular, margin entire, 11–12 × 9–10 mm; inner 1–3, widely ovate, 8–11 × 4–7 mm; ovary yellowish-green, trigonous-ellipsoid, 8.0–8.5 × 5.2–5.5 mm (wings excluded), sparsely pilose, 3-locular, upper 1 (abaxial wing side) degenerated occasionally; placentation axile, bilamellate. ***Styles*** 3, fused at base, yellow, ca. 3 mm in diameter, stigma spirally U-shaped twisted. ***Capsules*** nodding, trigonous-ellipsoid, 9–10 × 6.0–6.5 mm (wings excluded), yellowish-green, sparsely pilose, wings 3, unequal, 2-lateral wings smaller, crescent-shaped, abaxial wing semilune-shaped.

#### Distribution and habitat.

**Figure 3.**
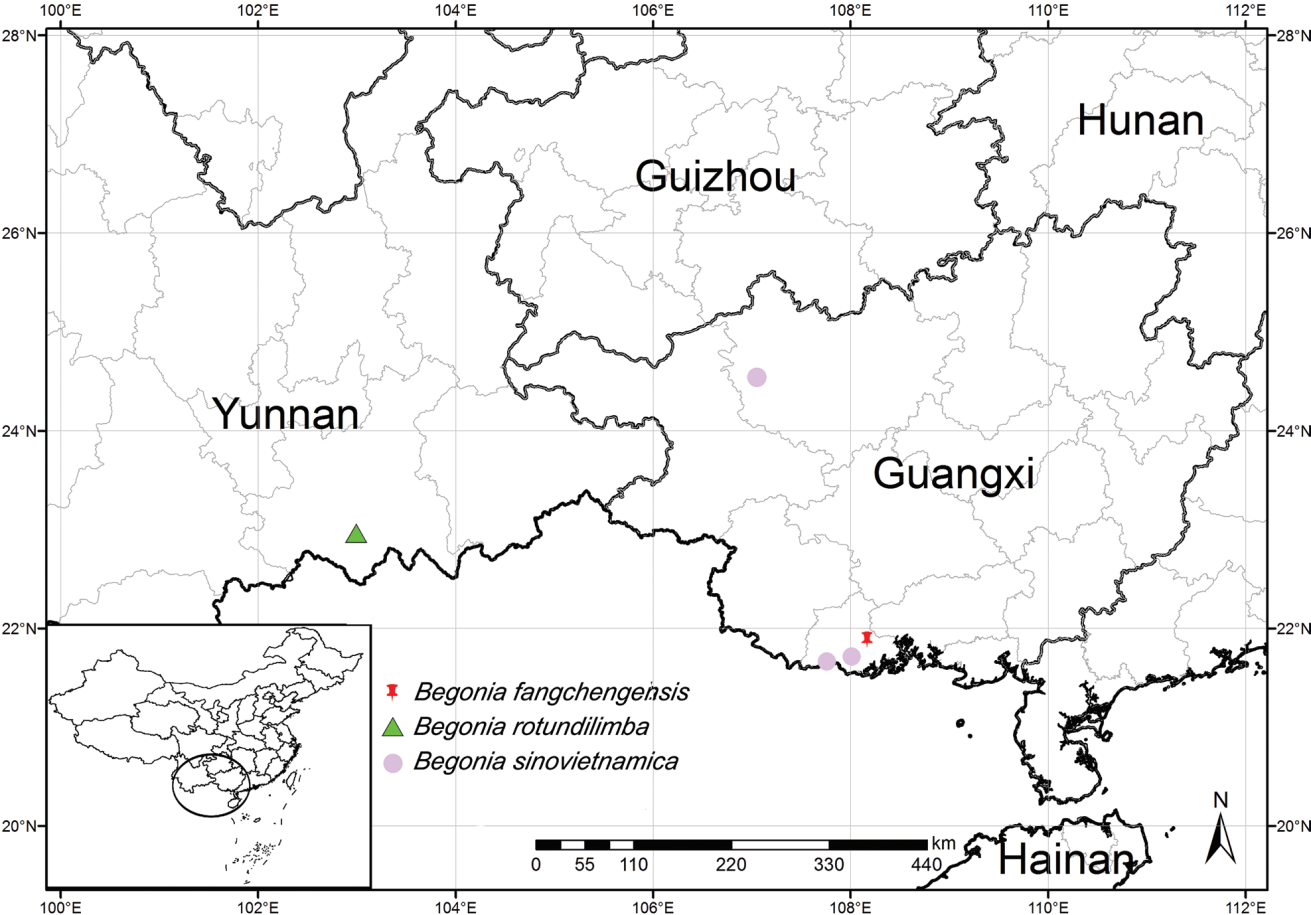
Distribution of *B.fangchengensis*, *B.rotundilimba* and *B.sinovietnamica*.

Currently only known from one locality in Fangcheng District, Fangchenggang City, Guangxi, China (Fig. 3). It grows in shaded environments along the stream or near a waterfall under the broad-leaved forest.

#### Phenology.

November-December (Flowering), January- February (Fruiting).

#### Etymology.

The specific epithet “*fangchengensis*” refers to the Fangcheng District, Guangxi where the species is distributed.

#### Conservation.

Only two populations with approximately 200 individuals have been found in the type locality. According to the IUCN Red List Categories and Criteria (IUCN 2024), *B.fangchengensis* should be assessed as ‘Endangered (D)’ due to its small geographic distribution and low population size.

## ﻿Discussion

The putative new *Begonia* species is morphologically characterized by having three styles, 3-locular ovary with axile and bifid placentation, representing the typical circumstance of the BegoniasectionDiploclinium (Lindl. 1846: 319) de Candolle (1859: 129). A total of 53 begonias of this section have been reported in China and most of them occur in Yunnan and Tibet, southeast China.

**Figure 4.**
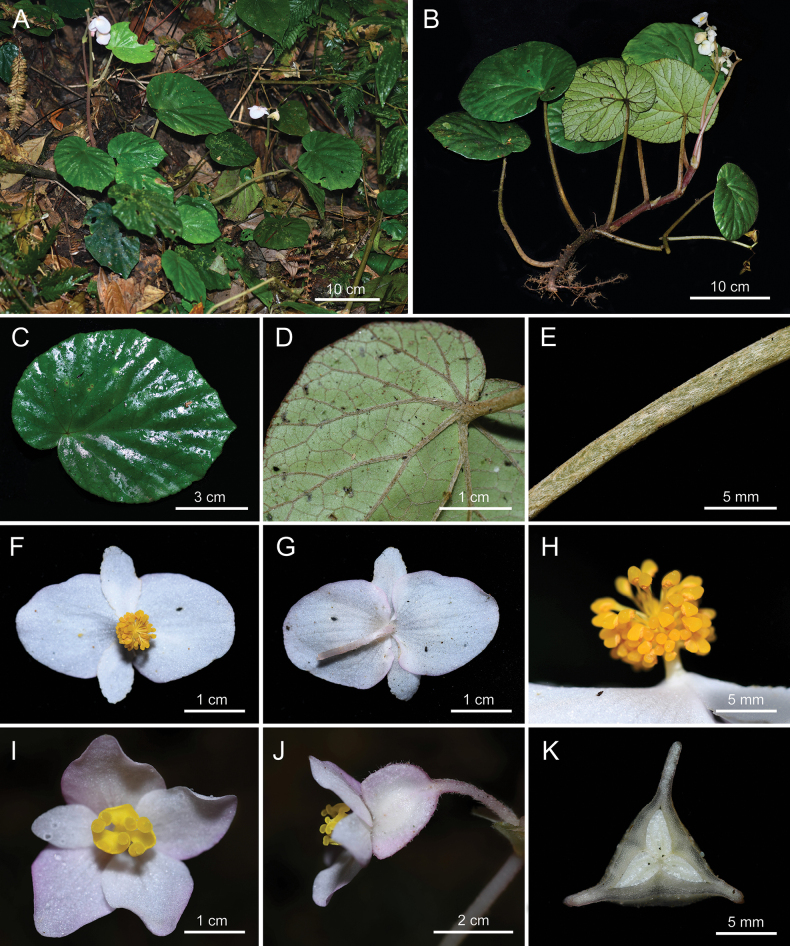
Habitat and morphology of *Begoniarotundilimba***A** habitat **B** flowering plant with erect stem at anthesis **C** leaf blade (adaxial) **D** leaf blade (abaxial) **E** petiole **F, G** front and back views of staminate flower **H** androecium **I, J** front and lateral view of pistillate flower **K** ovary section (Photos by W. G. WANG).

The putative new *Begonia* species mostly resembles *B.rotundilimba* from Pingbian, Yunnan in its elliptic leaf blade, palmate veins, dichasial cyme, three styles, unequally 3-winged capsule (Table 1, Fig. 4; Huang and Shui (1994)). In *B.rotundilimba*, there are erect stem at anthesis, green and villous petiole, and the stipule, leaf blade and inflorescence bract are all glabrous, being different from the creeping rhizome, pilose stipule and petiole, pubescent leaf blade and pilose inflorescence bract of *B.fangchengensis*. Furthermore, morphological dissimilarities also occur in reproductive organs, like the glabrous ovary and actinomorphic androecium of *B.rotundilimba*, compared with sparsely pilose ovary and the zygomorphic androecium in *B.fangchengensis*.

**Table 1.** Morphological comparisons of *B.fangchengensis* and relevant taxa.

Character	*B.rotundilimba*	*B.sinovietnamica*	*B.fangchengensis*
Rhizome	creeping, erect stem at anthesis; internode 2-3 cm long	elongate; internode 0.3-0.5 cm long	creeping; internode 0.3-1 cm long
Stipule	ovato-triangular, glabrous	oblong, pilose	triangular, pilose
Petiole	green, densely villous	reddish brown, densely villous	red, densely pilose
Leaves	blade adaxially green, glabrous	blade adaxially green, hirsute	blade adaxially bright green, smooth
Inflorescence bract	bracts glabrous, apex acuminate	bracts glabrous, apex acuminate	bracts apex 2-3 lobed, both pilose
Staminate flower	androecium actinomorphic, anthers oblong	anthers oblong, apex obtuse	androecium zygomorphic, anthers cuneiform, apex slightly bent and retuse
Pistillate flower	tepals 5, styles 3, ovary pilose, unequally 3-winged	tepals 4, styles 3, ovary sparsely pilose, subequally 3-winged	tepals 3-5, styles 3, ovary sparsely pilose, unequally 3-winged
Phenology (flowering; fruiting)	Apr; Jul	Jul; Aug	Nov; Jan
Habitat	alt. 1600-1800 m, Yunnan	alt. 230-400 m, Guangxi	alt. 198 m, Guangxi

Besides the similarity to *B.rotundilimba* in morphology, the putative new *Begonia* species is closest to *B.sinovietnamica* in geographic distribution and altitude (200–400 m) (Fig. 3). In *B.sinovietnamica*, leaf blade is adaxially hirsute, tepals of pistillate flower are 4 and the 3-winged ovary is subequal, in contrast with the adaxially smooth blade, 3–5 tepals of pistillate flower and unequally 3-winged ovary of the new species. Furthermore, the two species also differ from each other in phenology, including flowering and fruiting time (Table 1; Wu (1997)).

## Supplementary Material

XML Treatment for Begonia fangchengensis

## ﻿Additional information

### Author ORCIDs

Ping-Ting Hu https://orcid.org/0009-0001-2656-167X

Yu-Ni Huang https://orcid.org/0000-0001-5421-4192

Bi-Xuan Chen https://orcid.org/0009-0006-5022-6229

Xin-Xin Feng https://orcid.org/0000-0002-7421-2773

### Data availability

All of the data that support the findings of this study are available in the main text.
